# Associations between microRNA binding site SNPs in *FGFs* and *FGFRs* and the risk of non-syndromic orofacial cleft

**DOI:** 10.1038/srep31054

**Published:** 2016-08-11

**Authors:** Dandan Li, Hongchuang Zhang, Lan Ma, Yue Han, Min Xu, Zhendong Wang, Hongbing Jiang, Weibing Zhang, Lin Wang, Yongchu Pan

**Affiliations:** 1 Institute of Stomatology, Nanjing Medical University, Nanjing, 210029, China; 2 Jiangsu Key Laboratory of Oral Diseases, Nanjing Medical University, Nanjing, 210029, China; 3 Xuzhou First People’s Hospital, Xuzhou, 221000, China

## Abstract

We hypothesized that microRNA binding site single nucleotide polymorphisms (SNPs) in fibroblast growth factors (*FGFs*) and their receptor genes (*FGFRs*) may affect microRNA and mRNA interactions and are thereby associated with susceptibility of non-syndromic orofacial cleft (NSOC). Ten SNPs among the *FGF* and *FGFR* genes were selected and their associations with NSOC susceptibility were investigated in a case-control study of 602 patients with NSOC and 605 healthy controls. *FGF2*/rs1048201, *FGF5*/rs3733336 and *FGF9*/rs546782 showed suggestive association with NSOC susceptibility. In the combination analysis, the observed odds ratios (ORs) decreased with the number of protective alleles (rs1048201-T, rs3733336-G and rs546782-T) but were not statistically significant beyond the first comparison. Hsa-miRNA-496, hsa-miRNA-145 and hsa-miRNA-187 were predicted to be miRNAs with binding sites within/near these SNPs and were expressed in lip tissues. Decreased *FGF2*, *FGF5* and *FGF9* expression was observed in three cell lines transfected with the corresponding miRNAs. Moreover, the three SNPs could contribute to differential binding efficacy between hsa-miRNA-496 and *FGF2*, hsa-miRNA-145 and *FGF5*, hsa-miRNA-187 and *FGF9* in luciferase assay. The results suggest that *FGF2*/rs1048201, *FGF5*/rs3733336 and *FGF9*/rs546782 are associated with the risk of NSOC and that these miRNA-*FGF* interactions may affect NSOC development.

Non-syndromic orofacial cleft (NSOC) is the most common facial deformity and the main type of orofacial cleft. NSOC affects approximately one in seven hundred neonates worldwide with substantial ethnic and geographic variations^1^. NSOC can affect not only oral functions but also the health-related quality of life. The medical expenditures for NSOC are approximately decupled compared with unaffected children and also include the costs required for caregivers, dental care, speech therapy, and special education^2^. NSOC etiology involves more complicated heredity and environmental factors compared with the syndromic category^3^.

MicroRNAs (miRNAs) are endogenous non-coding RNAs that are approximately 20–25 nt in length. miRNAs play important roles in living organisms by pairing with mRNAs to guide their post-transcriptional expression and inhibit their translation, resulting in the destabilization of their target mRNAs^4^. miRNAs are widely expressed in developing murine embryonic orofacial tissues, and some play developmental roles by targeting genes involved in critical orofacial development processes, such as cell proliferation, apoptosis, cell adhesion, differentiation, and epithelial-mesenchymal transition (EMT)^5^. For instance, miRNA-140 is a critical regulator of the Pdgf signaling pathway during palatogenesis^6^ and the miRNA-17-92 cluster regulates the expression pattern of critical T-box transcriptional regulators during midface development^7^. Therefore, the miRNA-mRNA interaction is an important mechanism in orofacial development. However, this interaction can be affected by single nucleotide polymorphisms (SNPs) within or near the binding site (commonly referred to as miRNA binding site SNPs) that may alter the thermodynamic interplay between them^8^. For example, a mutation 10bp away from a predicted miRNA-140 binding site in the *PDGFRa* 3′-UTR resulted in increased binding affinity for miRNA-140 and thus depressed *PDGFRa* mRNA expression during human palate development^9^. Similarly, our previous study showed that the differential interaction between miR-3649 and its polymorphic target in the 3′-UTR of the muscle segment homeobox1 (*MSX1*) played a crucial role in NSOC susceptibility^10^.

Fibroblast growth factors (*FGFs*) and fibroblast growth factor receptors (*FGFRs*) have critical functions in orofacial development^11^. Highly conserved throughout metazoan evolution, these growth factors have been detected in thousands of animal species ranging from nematodes and zebrafish to mice and humans^12^. There are 18 mammalian *FGF* genes (*FGF1*–*FGF10*and *FGF16*–*FGF23*) and four primary *FGFR* genes (*FGFR1*–*FGFR4*) that encode ligands and receptors respectively, which initiate the FGF signaling cascade. These genes are especially known for their roles in the induction and migration of neural crest cells, skeletogenesis and EMT, all of which are fundamental processes for craniofacial development^13^. Aberrations in this pathway are associated with many types of craniofacial congenital disorders, including the syndromic forms of cleft lip and palate such as Kallman syndrome, Crouzon syndrome and Apert syndrome^14^. Furthermore, FGF signaling is closely integrated with other craniofacial development-related pathways, such as BMP, SHH, WNT, TGF and MSX^15^. Therefore, FGF signaling pathway has steady functions in lip and palate morphogenesis, with perturbation of its expression patterns sometimes leading to cleft pathogenesis^16^. To date, several studies have found that SNPs in *FGF*s and *FGFR*s are associated with NSOC development. For example, SNPs in *FGF1* were shown to be associated with a NSOC predisposition^16^,^17^. However, the majority of these studies were conducted in Caucasians with a limited sample size and none focused on miRNA binding site SNPs in *FGFs* and *FGFRs* that might also contribute considerable genetic contributions to NSOC. To address these issues, in this study we systematically selected miRNA-binding site SNPs in *FGFs* and *FGFRs* and investigated their associations with the risk of NSOC in a case-control study. Additionally, functional studies were performed to interpret the functions of the associated SNPs.

## Results

### Characteristics of the study subjects

The detailed characteristics of the study subjects are shown in Supplementary Table S1. There was no statistical difference in gender distribution between the cases and controls. The difference of age distributions is of no concern because oral clefts are birth defects, and the measurements of gene expressions are taken among the affected only. The case group could be divided into three subgroups according to the clinical phenotypes [cleft lip only (CLO), cleft lip with cleft palate (CLP) and cleft palate only (CPO), accounting for 39.5%, 50.6% and 9.3% of the cases, respectively].

### General SNP information

As shown in Table 1, ten candidate SNPs located in seven genes (*FGFR1*, *FGFR2, FGF2, FGF5, FGF6, FGF9* and *FGF17*) were identified in the study based on the inclusion criteria; their potential binding miRNAs are also listed. All of these SNPs were successfully genotyped with call rates greater than 95%. The genotype frequencies among the controls were consistent with the Hardy-Weinberg equilibrium (HWE, *P* > 0.05) with the exception of rs2241286 (*P* = 0.009) based on the preliminary analyses (Table 2).

## Association Studies

### Overall association analysis

After excluding rs2241286 from further analysis (deviation from HWE), nine SNPs remained in the association analysis with NSOC susceptibility. Allelic comparisons and genotypic comparisons including the heterozygous, homozygous and additive models were applied. Among them, three SNPs [rs1048201 on *FGF2* (*P*_add_ = 0.026), rs3733336 on *FGF5* (*P*_add_ < 0.001), and rs546782 on *FGF9* (*P*_add_ = 0.043)] showed significant associations with NSOC susceptibility in the additive model (Table 2). For rs1048201, a significant association was observed in all genetic models with the T allele associated with a reduced NSOC risk (OR_all_ = 0.83, 95% CI = 0.71–0.98; OR_het_ = 0.70, 95% CI = 0.13–0.54; OR_hom_ = 0.72, 95% CI = 0.17–0.52; OR_add_ = 0.83, 95% CI = 0.71–0.98). A protective effect of rs3733336-G was also found in all of the models (OR_all_ = 0.73, 95% CI = 0.60–0.88; OR_het_ = 0.74, 95% CI = 0.28–0.83; OR_hom_ = 0.48, 95% CI = 0.28–0.83; OR_add_ = 0.72, 95% CI = 0.59–0.87). A notable relationship between rs546782 and the risk of NSOC was observed in the allelic comparison (OR_all_ = 0.57, 95% CI = 0.33–0.98) and additive comparison model (OR_add_ = 0.57, 95% CI = 0.33–0.98).

### Stratified analysis

Appreciable differences were identified in the CLO, CLP and CPO etiology, which prompted us to conduct stratified analyses^18^. As shown in Supplementary Table S2, 232, 289 and 49 patients were included in the CLO, CLP and CPO groups, respectively. Protective effects of the rs1048201-T allele were observed for CLO and CLP (*P*_add_ = 0.028 for CLO and *P*_het_ = 0.046 for CLP) and the rs3733336-G allele for CLO and CPO (*P*_add_ = 0.012 for CLO and *P*_add_ = 0.017 for CPO). A lack of association was observed between rs546782 and any NSOC subgroups (Supplementary Table S3). The multinomial logistic regression of three subtypes showed the similar results by additive model (Supplementary Table S4).

### Combination analysis

Next, we evaluated the combined effects of the three SNPs that were significant in the overall analysis. The observed ORs decreased with the number of protective alleles (rs1048201-T, rs3733336-G and rs546782-T) but were not statistically significant beyond the first comparison. Individuals carrying one, two, and three to six protective alleles (rs1048201-T, rs3733336-G and rs546782-T) had a 0.67, 0.53 and 0.48-fold decreased risk of NSOC compared with individuals carrying no protective allele, respectively (*P* < 0.001 in the multi degree-of-freedom likelihood ratio test). However, there was no statistically significant difference of the NSOC risk between the groups carrying one, two, and three to six protective alleles (Table 3).

As shown in Supplementary Table S5, combined effects were also assessed in the three NSOC subgroups. Individuals with one protective allele were associated with a decreased risk of CLP. Associations with decreased CLO, CLP and CPO risks were observed with two or more protective alleles (*P* = 0.048 in CLO, *P* = 0.001 in CLP and *P* = 0.538 in CPO).

### Functional Studies

We subsequently conducted functional analysis on the three SNPs (rs1048201, rs3733336, and rs546782) that had suggestive associations with the NSOC risk in association studies.

### Lip tissue expression of the predicted binding miRNA partners

**Figure 1:**
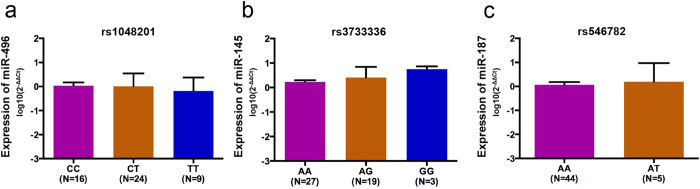
miRNA expression in lip tissues of non-syndromic orofacial cleft cases. (**a**–**c**) miR-496, miR-145 and miR-187 expression in 49 lip skin tissue samples from NSOC patients by qPCR, respectively. The results were normalized to *U6.* Error bars indicate the standard errors.

As predicted by the bioinformatics analysis and illustrated in Supplementary Figure S1, these three SNPs might affect the binding ability of hsa-miRNA-496 (miR-496), hsa-miRNA-145 (miR-145) and hsa-miRNA-187 (miR-187) to the corresponding *FGFs*. We collected redundant lip skin tissues from orofacial cleft plastic surgeries to confirm miRNA expression in lip tissues. As shown in Fig. 1a–c, these miRNAs were stably expressed in all lip tissues without association with the *FGF2*/rs1048201, *FGF5*/rs3733336 and *FGF9*/rs546782 genotypes, respectively.

### Confirmation of the interaction between the miRNAs and mRNAs *in vitro*

**Figure 2:**
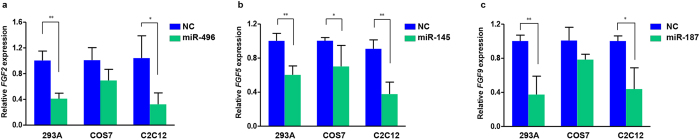
Quantitative real-time polymerase chain reaction analysis of *FGF2, FGF5 and FGF9* expression *in vitro*. (*P < 0.05, **P < 0.01, ***P < 0.001). (**a**) *FGF2* expression levels in HEK-293A cells, COS7 cells and C2C12 cells transfected with the miR-496 mimics and nonsense RNA fragments (NC). (**b**) *FGF5* expression levels in HEK-293A cells, COS7 cells and C2C12 cells transfected with the miR-145 mimics and nonsense RNA fragments (NC). (**c**) *FGF9* expression levels in HEK-293A cells, COS7 cells and C2C12 cells transfected with the miR-187 mimics and nonsense RNA fragments (NC).

We transfected miR-496, miR-145 and miR-187 mimics into three cell lines (HEK-293A, COS7 and C2C12) to test whether the miRNAs interacted with their corresponding *FGF*s. As shown in Fig. 2a, *FGF2* expression was significantly decreased in HEK-293A (*P* = 0.004) and C2C12 cell lines (*P* = 0.032) transfected with miR-496. Transfection of the miR-145 mimic into HEK-293A, COS7 and C2C12 cell lines resulted in significantly decreased *FGF5* expression (*P* = 0.010, 0.020 and 0.007, respectively) (Fig. 2b). *FGF9* expression was significantly decreased in HEK-293A (*P* = 0.009) and C2C12 cell lines (*P* = 0.019) transfected with miR-187 (Fig. 2c).

### miRNA-mRNA interactions directed by SNPs

The dual luciferase reporter assay was performed to test whether these three SNPs contributed to the different binding efficiencies between the miRNAs and mRNAs.

**Figure 3:**
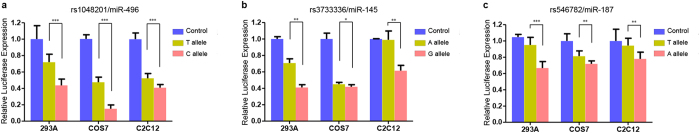
Binding ability assay of plasmids constructed with the 3′-UTR fragments of *FGF2*, *FGF5* and *FGF9* and the miRNAs in HEK-293A cells, COS7 cells and C2C12 cells. A plasmid construct with a nonsense sequence was used as the negative control (NC). The firefly luciferase to *Renilla* luciferase ratio was considered the relative luciferase expression. Independent triplicate experiments were performed. (*P < 0.05, **P < 0.01, ***P < 0.001). (**a**) The plasmid containing the C/T allele of rs1048201 was co-transfected with miR-496. (**b**) The plasmid containing the A/G allele of rs3733336 was co-transfected with miR-145. (**c**) The plasmid containing the A/T allele of rs546782 was co-transfected with miR-187.

The reporter gene assay suggested that the T allele of rs1048201 attenuated miR-496 binding in HEK-293A, COS7 and C2C12 cell lines compared with the C allele (*P* < 0.001 in all cell lines, Fig. 3a), whereas the G allele of rs3733336 had stronger binding affinity for miR-145 than the A allele in HEK-293A (*P* = 0.005), COS7 (*P* = 0.049) and C2C12 (*P* = 0.009) cell lines (Fig. 3b). The rs546782-T allele showed lower binding affinity for miR-187 compared with the A allele (*P* < 0.001 in HEK-293A, *P* = 0.001 in COS7, and *P* = 0.001 in C2C12 cell lines, Fig. 3c).

## Discussion

miRNA binding site SNPs can affect miRNA interactions with their putative target sites due to allele differences. This type of influence may be functional and alter the expression level of the target genes, thereby contributing to altered phenotypes^19^. In the present study, ten miRNA-binding site SNPs in seven *FGF*/*FGFR* genes were selected and successfully genotyped in a case-control study. One of the SNPs (rs2241286) was excluded from the analysis due to its deviation from the HWE. Of the remaining nine SNPs, three SNPs (*FGF2*/rs1048201, *FGF5*/rs3733336, and *FGF9*/rs546782) exhibited evidence of association with NSOC in the Chinese Han population. Additionally, *FGF2*/rs1048201 and *FGF5*/rs3733336 could be favorable indicators of an individual’s susceptibility to specific subgroups. The combined analysis showed that the protective effects against NSOC strengthened with the increasing number of protective alleles.

The protective T allele of rs1048201 decreased the *FGF2* binding capacity for miR-496 *in vitro* compared with the C allele, resulting in a higher mRNA level. These results corroborated the increased expression of *FGF2* during normal cranial fusion^20^. *FGF2* was demonstrated to have profitable effects on cell proliferation and angiogenesis in periodontal tissues and oral mucosa^21^ as well as cranial suture fusion^22^. Mutations contributing to impaired transcription in the non-coding region of *FGF2* were associated with the development of orofacial clefts^23^. Furthermore, *FGF2* treatment promoted rat osteoblast attachment and produced enhanced suture fusion in calvarial organ culture^24^.

*FGF5* has been recognized as a development-related gene. It is widely expressed in embryos and participates in the interaction between the epithelium and mesenchymal cells, the formation of the neural crest, angiogenesis and cell proliferation^13^,^25^. In the present study, rs3733336 in the *FGF5* 3′-UTR was identified as a functional SNP associated with the risk of NSOC. The G allele was recognized as a protective allele in the association study. The initially predicted hsa-miRNA-23a (miR-23a) (Table 1) was not demonstrated to bind *FGF5* as predicted (Supplementary Figure S2). We found that there was a binding site for hsa-miRNA-145 (miR-145) at 30bp upstream of rs3733336 in further analysis by Targetscan ( http://www.targetscan.org) and SNPinfo Web Server ( http://manticore.niehs.nih.gov/snpinfo/snpfunc.htm). We hypothesized that rs3733336 may affect interaction between miR-145 and *FGF5*. Finally, the luciferase reporter assay showed that the transcriptional activity of the reporter gene with the G allele was significantly reduced compared with the A allele in all three cell lines, indicating that the mutant allele in the 3′-UTR activated *FGF5* degradation as a protection mechanism. Thus, the interaction between rs3733336-G and miR-145 acted as a possible profitable mechanism to reduce the risk of NSOC.

*FGF9* regulated cell proliferation in the palatal mesenchyme during mouse palatogenesis and exhibited an extremely high expression level in the epithelial-mesenchymal interaction phase of the mouse embryo palate processes^26^. Similar to *FGF2*, an increased *FGF9* level is profitable. For instance, a total of 40% of *Fgf9*^−/−^ mouse embryos exhibited cleft palates in the study of Colvin *et al.*^27^. SNPs in the *FGF9* 3′-UTR were identified as critical markers for gene-gene interactions in NSOC etiology^16^. However, the biological mechanism has not been clarified to date. In the present study, the T allele of rs546782 broke down the pairing with miR-187 and exhibited a protective effect against NSOC.

The experimental design and potential mechanism underlying the actions by which these *FGF* and *FGFR* SNPs contribute to NSOC is illustrated in Supplementary Figure S3. Taken together, our findings demonstrate that *FGF2*/rs1048201, *FGF5*/rs3733336 and *FGF9*/rs546782 individually and jointly contribute to the risk of NSOC and the NSOC subgroups. These SNPs affected the binding ability of miRNAs to their target genes and then modified the expression levels of the *FGF* genes to contribute to NSOC susceptibility. These studies help illustrate the mechanism underlying NSOC. However, two major limitations should be addressed. First, we did not perform multiple corrections in the present study, which might contribute to false positive results. Additional replication studies are needed to verify our findings. Second, further investigations between these three SNPs and the risk of CPO are warranted due to the limited CPO sample size in the current study. Therefore, extensive studies are warranted to obtain a greater understanding of our findings.

## Material and Methods

### Sample recruitment

This study is an ongoing study approved by the Ethics Review Committee of Nanjing Medical University (NJMUERC [2008] No. 20). All the methods were carried out in accordance with the protocols approved by the ethics committee. All subjects in both groups voluntarily joined this study and provided informed consent. The present study consisted of 602 NSOC cases and 605 gender-matched healthy controls recruited from three affiliated hospitals of Nanjing Medical University (the Stomatological Hospital of Jiangsu Province, Xuzhou First People’s Hospital, and Nanjing Children’s Hospital) between August 2008 and August 2012^28^. All patients were clinically assessed according to the detailed diagnostic information obtained from the medical records and physical examinations. Only patients who had an isolated oral cleft without syndromic symptoms or other major congenital defects were included. The controls were examined by two experienced oral surgeons to ensure that they had no cleft, hypodontia or other congenital anomalies. Approximately 3ml of venous blood was drawn from each participant for the genotyping analysis.

### SNP selection

Common SNPs (minor allele frequency, MAF ≥ 5%) of 18 *FGFs* (*FGF1*–*FGF10* and *FGF16*–*FGF23*) and 4 *FGFR*s (*FGFR*1-*FGFR*4) of Homo sapiens in Chinese Han population based on the dbSNP database ( http://www.ncbi.nlm.nih.gov/projects/SNP/index.html) and HapMap Project database ( http://hapmap.ncbi.nlm.nih.gov/) were screened from gene regions (including ± 2kb). Then, *in silico* bioinformatics predictions from the SNPinfo Web Server ( http://manticore.niehs.nih.gov/snpinfo/snpfunc.htm) and mirSNP ( http://bioinfo.bjmu.edu.cn/mirsnp/search/) were collectively applied to select the miRNA binding site SNPs. Linkage disequilibrium (LD) analysis with an r^2^ threshold of 0.80 was applied to filter these SNPs. Finally, ten SNPs were analyzed in our study. The characteristic information of the final identified SNPs as well as their corresponding potential binding miRNAs are listed in Table 1.

### DNA extraction and genotyping

The conventional phenol-chloroform method was conducted to extract genomic DNA as previously described^10^,^28^. All samples with combinations of two negative and two positive references were genotyped using a blind method based on double ligation and multiplex fluorescence polymerase chain reaction (PCR) with a custom-by-design 2 × 48-Plex SNPscan^TM^ Kit with the support of Genesky Biotechnologies Inc. (Shanghai, China)^29^. A total of 5% of the samples were randomly repeated for quality control, and the results were totally concordant.

### Quantitative PCR of predicted miRNAs in lip skin tissues

A total of 49 redundant lip skin tissues of cleft cases were obtained from plastic surgeries and frozen in liquid nitrogen. Total RNA was extracted by the conventional TRIzol method (Invitrogen, Carlsbad, CA, USA) according to the manufacturer’s instructions. The miRNA expression levels were measured by quantitative PCR (qPCR) with the SYBR Green Real-Time PCR Master Mix kit (Takara, Shiga, Japan) on an ABI 7900 System (Applied Biosystems, Foster City, CA, USA) using the transcription level of *U6* as the internal control^30^ (Primers in Supplementary Table S6). The relative gene expression was quantified by the comparative Ct method also referred to as the 2^−ΔΔCt^ method^31^. All PCR assays were performed in triplicate to verify the results.

### Cell culture

HEK-293A, COS7 and C2C12 cell lines were purchased from the Shanghai Institute of Biochemistry and Cell Biology, Chinese Academy of Sciences (Shanghai, China), and cultured in Dulbecco's modified Eagle’s medium (Gibco, Foster City, CA, USA) with 10% fetal bovine serum (Gibco).

### miRNA transfection and qPCR

Forty-eight hours after transfection with the miRNA mimics using Lipofectamine 2000 (Invitrogen, Carlsbad, CA, USA) as recommended by the manufacturer, the cells were harvested for qPCR analysis.

Total RNA was extracted from the cells using the conventional TRIzol method (Invitrogen) according to the manufacturer’s instruction. The *FGF2*, *FGF5* and *FGF9* expression levels were measured by qPCR with the SYBR Green Real-Time PCR Master Mix kit (Takara, Shiga, Japan) on ABI 7900 System (Applied Biosystems). The *GAPDH* transcription level was detected as the internal control (Primers in Supplementary Table S6). All PCR assays were performed in triplicate to verify the results.

### Luciferase reporter plasmid construction

The *FGF2, FGF5* and *FGF9* 3′-UTR fragments containing the major alleles of SNPs were inserted downstream of the luciferase gene between two restrictive sites in the luciferase reporter psiCHECK^TM^-2 vector (Promega, Madison, WI, USA). The minor alleles were generated by the site-specific mutagenesis method using constructs containing the major alleles as the template. The accuracy of the recombinant plasmids was verified by DNA sequencing (the sequencing results of the fragments are shown in Supplementary Figure S4, ABC). The psiCHECK^TM^-2 vector with a random inserted fragment was applied as the negative control.

### Transient co-transfection and dual-luciferase reporter assay

Plasmids containing the wild type or mutant type allele were co-transfected with the miRNA mimics using Lipofectamine 2000 (Invitrogen). The luciferase activity in the lysates was quantified forty-eight hours after transfection with a dual-luciferase reporter assay system (Promega). The luminescent reaction of the *Renilla* luciferase was simultaneously activated after the firefly luciferase reporter was measured as a stabilized luminescent signal. The firefly luciferase to *Renilla* luciferase ratio was considered the relative reporter activity. Independent triplicate experiments were performed for each plasmid construct.

### Statistical analysis

Pairwise linkage disequilibrium (LD) was computed as r^2^ for all SNPs by the Haploview program. Student’s *t* test was employed for continuous values. The gender distribution between cases and controls was evaluated with a two-sided Chi-square test, and the HWE among the controls was calculated using a goodness-of-fit Chi-square test. All results were two-sided. *P* < 0.05 was considered statistically significant. All tests were performed with version 9.1 of the SAS^®^ software (SAS Institute Inc., Cary, NC, USA).

The association between the SNPs and the risk of NSOC and subgroups was measured by the odds ratio (OR) and 95% confidence interval (95% CI)^32^,^33^. Four kinds of genetic models were adopted. In Table 2, if T is the variant of interest (the mutant allele), and C is the dominant allele, a 2 by 2 table and an unconditional logistic regression with one degree of freedom could be used to determine statistical significance of allele frequencies under the assumption of an allelic comparison^33^. A heterozygous comparison presented the comparison of CT genotypes with CC genotypes. Likewise, comparison of CC with TT genotypes was assumed as homozygous comparison. Scores of 0, 1, and 2 assigned to genotype CC, CT, and TT respectively and ORs calculated by unconditional logistic regression model was a test for association between the variant allele and the disease named as additive model.

## Additional Information

**How to cite this article**: Li, D. *et al.* Associations between microRNA binding site SNPs in *FGFs* and *FGFRs* and the risk of non-syndromic orofacial cleft. *Sci. Rep.* **6**, 31054; doi: 10.1038/srep31054 (2016).

## Supplementary Material

Supplementary Information

## Figures and Tables

**Table 1:** Primary information of selected SNPs.

ID	rs No.	Chr	Position	Gene	Allele	MAF in CHB	Predicted miRNAs^a^
1	rs13317	8	38388671	*FGFR1*	C > T	T: 0.427	has-miR-3128
2	rs1476215	4	124037721	*FGF2*	T > A	A: 0.089	hsa-miR-196a
3	rs1048201	4	124033758	*FGF2*	C > T	T: 0.375	hsa-miR-496
4	rs1047057	10	123229102	*FGFR2*	G > A	A: 0.375	hsa-miR-575, hsa-miR-770-5p
5	rs4690150	4	81430594	*FGF5*	C > G	G: 0.387	hsa-miR-452
6	rs6838203	4	81428016	*FGF5*	T > A	A: 0.337	hsa-miR-1243, hsa-miR-509-3p
7	rs3733336	4	81426987	*FGF5*	A > G	G: 0.253	hsa-miR-23a
8	rs2241286	12	4413622	*FGF6*	G > A	A: 0.232	hsa-miR-4677-3p
9	rs546782	13	21173946	*FGF9*	A > T	T: 0.083	hsa-miR-187
10	rs3176304	8	21961718	*FGF17*	G > C	C: 0.275	hsa-miR-324-3p

Chr, Chromosome; MAF, minor allele frequency; CHB, Chinese Han Beijing.

^a^SNPinfo Web Server ( http://manticore.niehs.nih.gov/snpinfo/snpfunc.htm) and mirSNP ( http://bioinfo.bjmu.edu.cn/mirsnp/search/).

**Table 2:** Association of SNPs with non-syndromic orofacial cleft susceptibility.

SNP ID	*P* value for HWE	Allelic Comparison		Co-dominant model				Additive model	
		*P*_all_^a^	OR, 95%CI	*P*_het_^b^	OR, 95%CI	*P*_hom_^c^	OR, 95%CI	*P*_add_^d^	OR, 95%CI
rs13317	0.474	0.935	0.99 [0.84, 1.18]	0.919	1.01 [0.80, 1.29]	0.859	0.97 [0.66, 1.42]	0.934	0.99 [0.84, 1.18]
rs1476215	0.522	0.414	1.17 [0.80, 1.70]	0.302	1.23 [0.83, 1.81]	0.999	—	0.406	1.18 [0.80, 1.72]
**rs1048201**	1.000	**0.026**	**0.83 [0.71, 0.98]**	**0.007**	**0.70 [0.13, 0.54]**	**0.049**	**0.72 [0.17, 0.52]**	**0.026**	**0.83 [0.71, 0.98]**
rs1047057	0.596	0.342	1.08 [0.92, 1.27]	0.194	1.19 [0.92, 1.53]	0.450	1.14 [0.81, 1.59]	0.337	1.08 [0.92, 1.28]
rs4690150	0.253	0.49	0.94 [0.80, 1.11]	0.785	0.97 [0.75, 1.25]	0.457	0.88 [0.18, 0.62]	0.482	0.94 [0.80, 1.11]
rs6838203	0.852	0.469	1.06 [0.90, 1.26]	**0.039**	**1.30 [1.01, 1.67]**	0.952	0.99 [0.69, 1.42]	0.468	1.06 [0.90, 1.26]
**rs3733336**	0.181	**0.001**	**0.73 [0.60, 0.88]**	**0.013**	**0.74 [0.28, 0.83]**	**0.008**	**0.48 [0.28, 0.83]**	** < 0.001**	**0.72 [0.59, 0.87]**
rs2241286	0.009	—	—	—	—	—	—	—	—
**rs546782**	0.509	**0.040**	**0.57 [0.33, 0.98]**	0.066	0.60 [0.34, 1.04]	—	—	**0.043**	**0.57 [0.33, 0.98]**
rs3176304	1.000	0.689	1.04 [0.86, 1.26]	0.287	1.14 [0.90, 1.45]	0.613	0.88 [0.53, 1.46]	0.689	1.04 [0.86, 1.26]

SNP, single nucleotide polymorphism; Chr, chromosome; OR, odds ratio; 95%CI, 95% confidence interval.

^a^*P* value of allelic comparison; rs1048201- T *vs*. C, rs3733336- G *vs.* A, rs546782-T *vs.* A.

^b^*P* value of heterozygous comparison; rs1048201 - CT *vs*. CC, rs3733336 - AG *vs.* AA, rs546782 - AT *vs.* AA.

^c^*P* value of homozygous comparison; rs1048201 - TT *vs*. CC, rs3733336 - GG *vs.* AA, rs546782-TT *vs.* AA.

^d^*P* value of additive model analysis.

**Table 3:** Combined effects of three SNPs on non-syndromic orofacial cleft.

Protective allele number^a^	Case (%)	Control (%)	OR (95%CI)	*P*
0	118 (20.59)	77 (13.05)	Reference	
1	220 (38.39)	214 (36.27)	**0.67 (0.48–0.95)**	**0.023**
2	170 (29.67)	210 (35.59)	**0.53 (0.37–0.75)**	<**0.001**
3–6	65 (11.34)	89 (15.08)	**0.48 (0.31–0.73)**	<**0.001**
1–6	455 (79.41)	513 (86.95)	**0.58 (0.42–0.79)**	<**0.001**
*P*^b^ < 0.001				
2 *vs.* 1	—	—	0.78 (0.59–1.03)	0.083
3–6 *vs.* 2	—	—	0.91 (0.62–1.33)	0.653
3–6 *vs.* 1	—	—	0.71 (0.49–1.04)	0.081

^a^rs1048201-T, rs3733336-G and rs546782-T were assumed as protective alleles based on the association study in Table 2.

^b^*P* value of multi degree-of-freedom likelihood ratio test.
