# Supplementary material for: Associations between microRNA binding site SNPs in FGFs and FGFRs and the risk of non-syndromic orofacial cleft
Source: Sci Rep. 2016 Aug 11;6:31054. doi: 10.1038/srep31054 (PMC4980626; doi:10.1038/srep31054)
Supplement: Supplementary Information [file srep31054-s1.pdf]

Associations between microRNA binding site SNPs in *FGFs* and *FGFRs* and the risk of non-syndromic orofacial cleft

Dandan Li[1,2], Hongchuang Zhang[3], Lan Ma[1,2], Yue Han[1,2], Min Xu[1,2], Zhendong Wang[1,2], Hongbing Jiang[1,2], Weibing Zhang[1,2], Yongchu Pan[1, 2*], Lin Wang[1,2*]

1 Institute of Stomatology, Nanjing Medical University, Nanjing, 210029, China,

2 Jiangsu Key Laboratory of Oral Diseases, Nanjing Medical University, Nanjing, 210029, China

3 Xuzhou First People's Hospital, Xuzhou, 221000, China

*Corresponding authors, nydwlktz@gmail.com (LW) and panyongchu@njmu.edu.cn (YP)

Table S1. Demographic characteristic information of cases and controls

Variables	Cases (N=602)		Controls (N=605)	
	N	%	N	%
Age[a]	1.0 (0.5, 3.0)	-	9.1(8.7, 9.7)	-
Gender				
Male	372	61.8	362	60.1
Female	230	38.2	243	39.9
Subgroups				
CLO	238	39.5		
CLP	305	50.6		
CPO	56	9.3		

CLO, cleft lip only; CLP, cleft lip and palate; CPO, cleft palate only

[a] Median (Percentiles 25, Percentiles 75)

Table S2. Stratified distribution of study subjects by subtypes of deformity

Genotype	Controls (n=593, %)	CLO (n=232, %)	CLP (n=289, %)	CPO (n=49, %)
**rs1048201**				
CC	158 (26.6)	81 (34.9)	96 (33.2)	16 (32.7)
CT	310 (52.3)	110 (47.4)	134 (46.4)	25 (51.0)
TT	125 (21.1)	41 (17.7)	59 (20.4)	8 (16.3)
**rs3733336**				
AA	308 (52.2)	139 (59.9)	170 (58.8)	34 (69.4)
GA	246 (41.7)	86 (37.1)	105 (36.3)	14 (28.6)
GG	39 (6.6)	7 (3.0)	14 (4.8)	1 (2.0)
**rs546782**				
AA	558 (94.1)	223 (96.1)	278 (96.2)	49 (100.0)
AT	34 (5.7)	9 (3.9)	11 (3.8)	0 (0.0)
TT	1 (0.2)	0 (0.0)	0 (0.0)	0 (0.0)

CLO, cleft lip only; CLP, cleft lip and palate; CPO: cleft palate only

Table S3. Stratified analysis by subtypes of deformity

Phenotype	Analysis model	rs1048201		rs3733336		rs546782	
		OR (95% CI)	*P*	OR (95% CI)	*P*	OR (95% CI)	*P*
CLO							
	Allelic comparison[a]	**0.78 (0.63-0.97)**	**0.028**	**0.70 (0.53-0.92)**	**0.010**	0.65 (0.31-1.35)	0.254
	Heterozygous comparison[b]	**0.69 (0.49-0.98)**	**0.038**	0.77 (0.56-1.06)	0.105	0.63 (0.32-1.44)	0.311
	Homozygous comparison[c]	**0.63 (0.40-0.99)**	**0.043**	**0.39 (0.17-0.91)**	**0.028**	—	0.982
	Additive model	**0.78 (0.63-0.97)**	**0.028**	**0.71 (0.55-0.93)**	**0.012**	0.65 (0.31-1.36)	0.255
CLP							
	Allelic comparison[a]	0.88 (0.72-1.07)	0.172	0.79 (0.62-1.00)	0.053	0.61 (0.30-1.19)	0.142
	Heterozygous comparison[b]	**0.72 (0.52-0.99)**	**0.046**	0.78 (0.58-1.05)	0.100	0.63 (0.31-1.26)	0.187
	Homozygous comparison[c]	0.79 (0.53-1.18)	0.248	0.65 (0.34-1.23)	0.181	—	0.981
	Additive model	0.87 (0.71-1.06)	0.170	0.79 (0.62-1.00)	0.053	0.60 (0.30-1.19)	0.142
CPO							
	Allelic comparison[a]	0.79 (0.52-1.22)	0.286	**0.50 (0.29-0.88)**	**0.017**	—	—
	Heterozygous comparison[b]	0.79 (0.41-1.52)	0.479	**0.51 (0.27-0.98)**	**0.043**	—	—
	Homozygous comparison[c]	0.63 (0.26-1.53)	0.309	0.23 (0.03-1.71)	0.150	—	—
	Additive model	0.79 (0.52-1.22)	0.286	**0.50 (0.29-0.88)**	**0.017**	—	—

CLO, cleft lip only; CLP, cleft lip and palate; CPO, cleft palate only

[a] Allelic comparison; rs1048201- T *vs*. C, rs3733336- G *vs.* A, rs546782-T *vs.* A

[b] Heterozygous comparison; rs1048201 - CT *vs*. CC, rs3733336 - AG *vs.* AA, rs546782 - AT *vs.* AA.

[c] Homozygous comparison; rs1048201 - TT *vs*. CC, rs3733336 - GG *vs.* AA, rs546782-TT *vs.* AA.

Table S4. Multinomial logistic regression of deformity subtypes with additive model.

Phenotype	rs1048201		rs3733336		rs546782	
	OR (95% CI)	*P*	OR (95% CI)	*P*	OR (95% CI)	*P*
CLO	**0.78 (0.63-0.97)**	**0.028**	**0.71 (0.55-0.93)**	**0.012**	0.62 (0.29-1.28)	0.196
CLP	0.85 (0.70-1.04)	0.115	**0.77 (0.60-0.97)**	**0.027**	0.64 (0.33-1.24)	0.185
CPO	0.93 (0.62-1.39)	0.723	**0.50 (0.29-0.88)**	**0.013**	—	—

CLO, cleft lip only; CLP, cleft lip and palate; CPO, cleft palate only

Table S5. Combined effect of three SNPs in three subtypes

Protective allele number [a]	CLO		CLP		CPO	
	OR (95%CI) [a]	*P*	OR (95%CI) [a]	*P*	OR (95%CI) [a]	*P*
0	Reference		Reference		Reference	
1	0.86 (0.55-1.35)	0.523	**0.56 (0.37-0.85)**	**0.006**	0.53 (0.25-1.11)	0.094
2	**0.59 (0.37-0.94)**	**0.028**	**0.51 (0.33-0.77)**	**0.001**	**0.37 (0.16-0.83)**	**0.015**
3-6	**0.43 (0.24-0.79)**	**0.007**	**0.55 (0.33-0.91)**	**0.019**	**0.27 (0.08-0.85)**	**0.025**
1-6	0.68 (0.45-1.02)	0.064	**0.54 (0.37-0.78)**	**0.001**	**0.42 (0.21-0.82)**	**0.010**
P^b	**0.048**		**0.001**		0.538	
2 *vs.* 1	0.78 (0.59-1.03)	0.083	0.68 (0.47-1.00)	0.050	0.70 (0.34-1.45)	0.334
3-6 *vs.* 2	0.91 (0.62-1.33)	0.653	0.72 (0.41-1.25)	0.248	0.73 (0.23-2.29)	0.585
3-6 *vs.* 1	0.71 (0.49-1.04)	0.081	**0.49 (0.29-0.83)**	**0.008**	0.51 (0.17-1.53)	0.228

[a] rs1048201-T, rs3733336-G and rs546782-T were assumed as protective alleles based on the association study in Table 2.

[b] *P* value of multi degree-of-freedom likelihood ratio test.

Table S6. Primers of microRNAs for quantitative PCR

**Gene Name**	Forward（5'-3'）	Reverse（5'-3'）
miR-496	ACACTCCAGCTGGGTGAGTATTACATGGCC	TGGTGTCGTGGAGTCG
miR-23a	ACACTCCAGCTGGGATCACATTGCCAGGG	TGGTGTCGTGGAGTCG
miR-145	ACACTCCAGCTGGGGUCCAGUUUUCCCAGGA	TGGTGTCGTGGAGTCG
miR-187	ACACTCCAGCTGGGTCGTGTCTTGTGTTGC	TGGTGTCGTGGAGTCG
U6	CTCGCTTCGGCAGCACA	AACGCTTCACGAATTTGCGT
FGF2	AGAAGAGCGACCCTCACATCA	CGGTTAGCACACACTCCTTTG
FGF5	CACTGATAGGAACCCTAGAGGC	CAGATGGAAACCGATGCCC
FGF9	GGCCTGGTCAGCATTCGAG	GTATCGCCTTCCAGTGTCCAC
GAPDH	GCACCGTCAAGGCTGAGAAC	TGGTGAAGACGCCAGTGGA

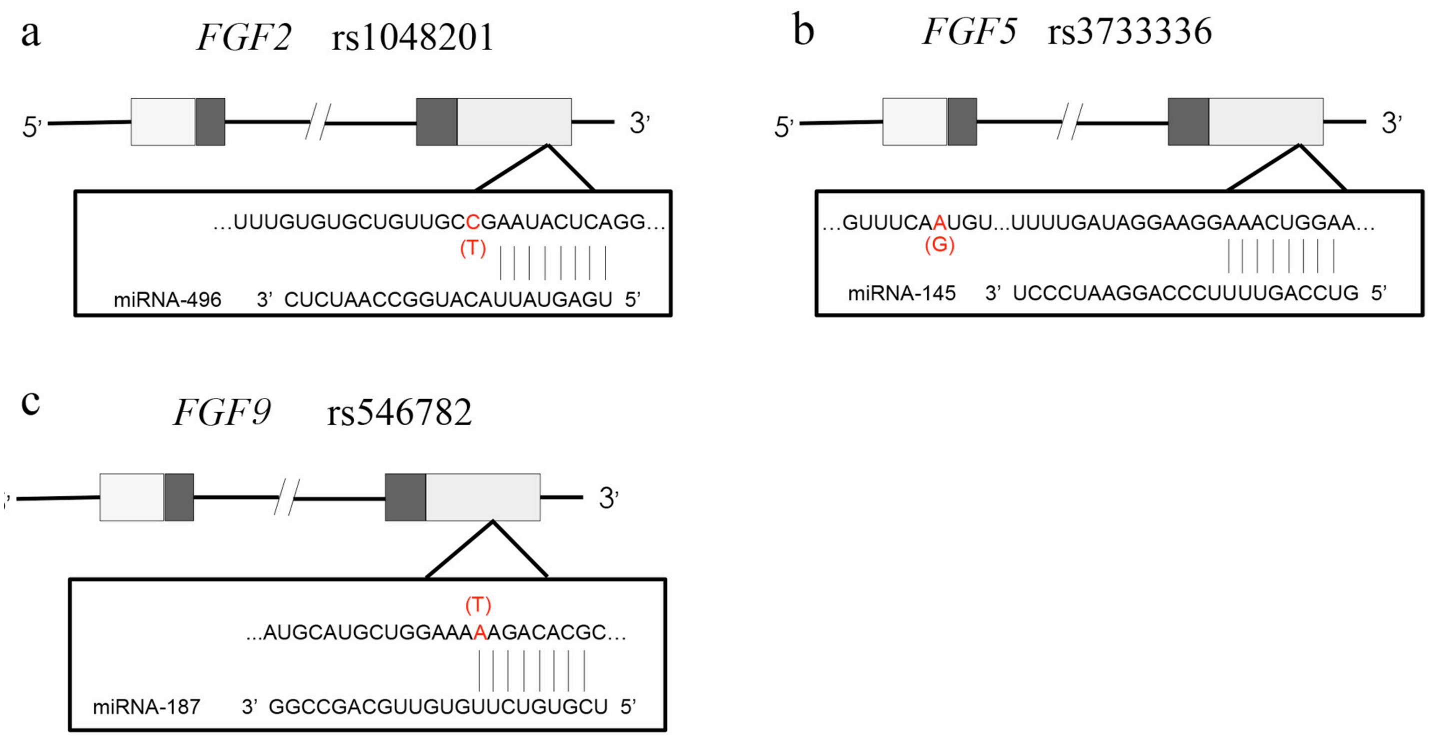

Figure S1. The presumed miRNA binding sequences.

(a) The putative binding of miR-496 and *FGF2* mRNA.

(b) The putative binding of miR-145 and *FGF5* mRNA.

(c) The putative binding of miR-187 and *FGF9* mRNA.

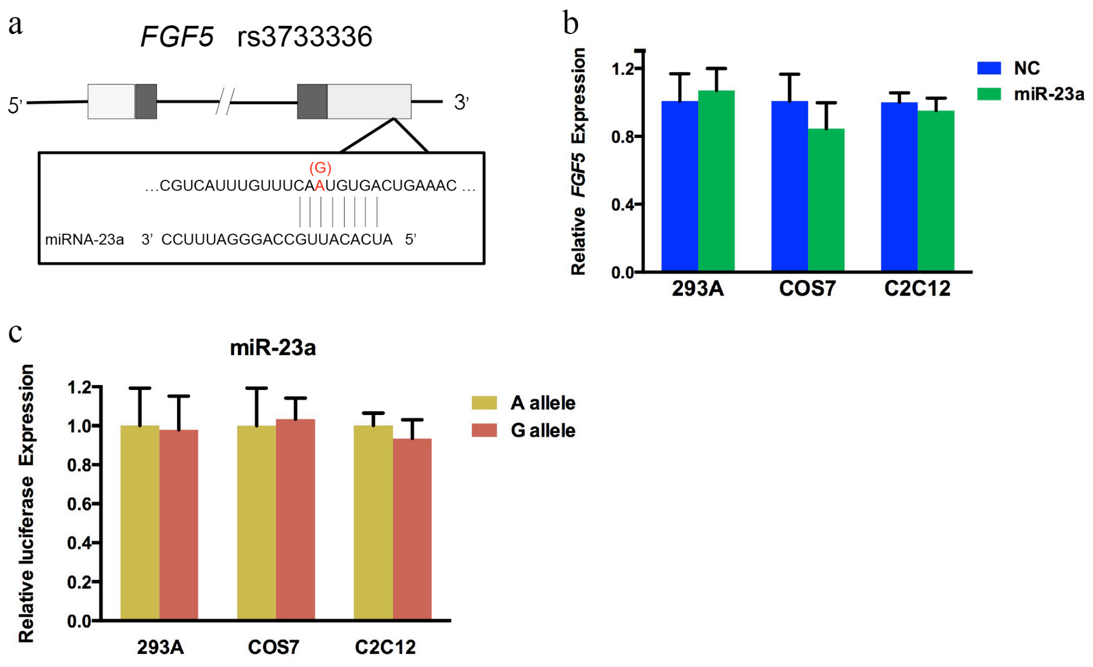

Figure S2. The binding ability test of miR-23a and 3'-UTR of *FGF5*.

miR-23a was predicted to have the binding ability with *FGF5* mRNA (**Figure S2, a**). However, *FGF5* expression was not affected by miR-23a mimics in all kinds of cells (**Supplementary Figure S2, b**). At the same time, rs3733336 was unable to affect miR-23a and *FGF5* interaction regarding the similar luciferase expressions between two alleles (**Supplementary Figure S2, c**). Therefore, miR-23a was demonstrated to not bind *FGF5* as predicted.

(a) The putative binding of miR-23a and *FGF5* mRNA.

(b) *FGF5* expression levels in HEK-293A cells、COS7 cells and C2C12 cells transfecting miR-23a mimics and nonsense RNA fragments (NC) respectively.

(c) Binding ability assay of plasmids construct with 3'-UTR fragment of *FGF5* and miR-23a in HEK-293A cells, COS7 cells and C2C12 cells. Ratio of Firefly luciferase to *Renilla* luciferase was considered as relative luciferase expression. Independent triplicate experiments were performed.

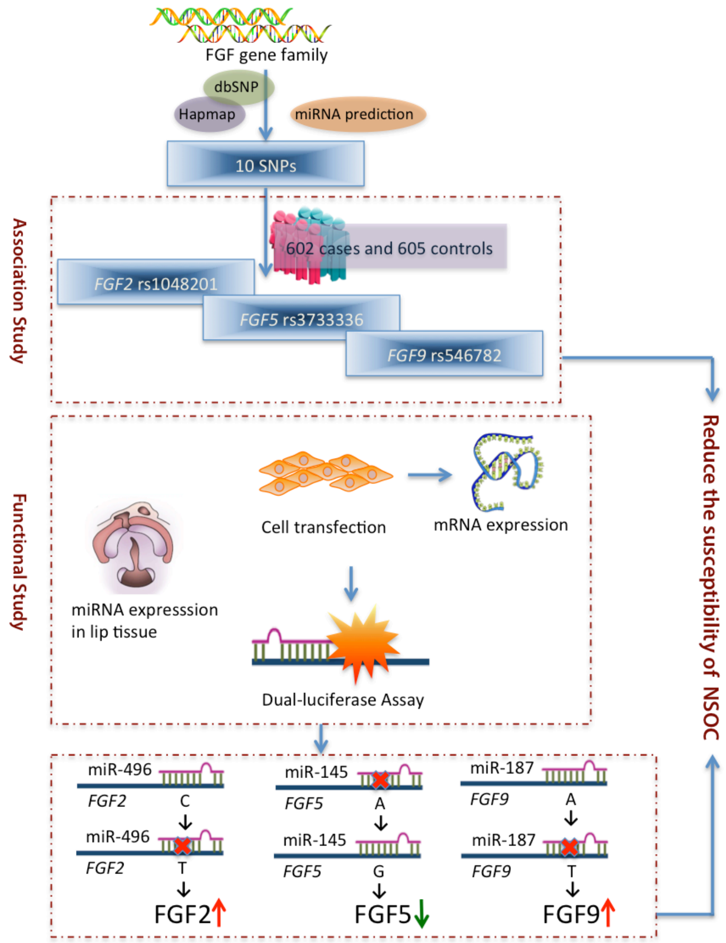

Figure S3. Sketch map of the experiment process.

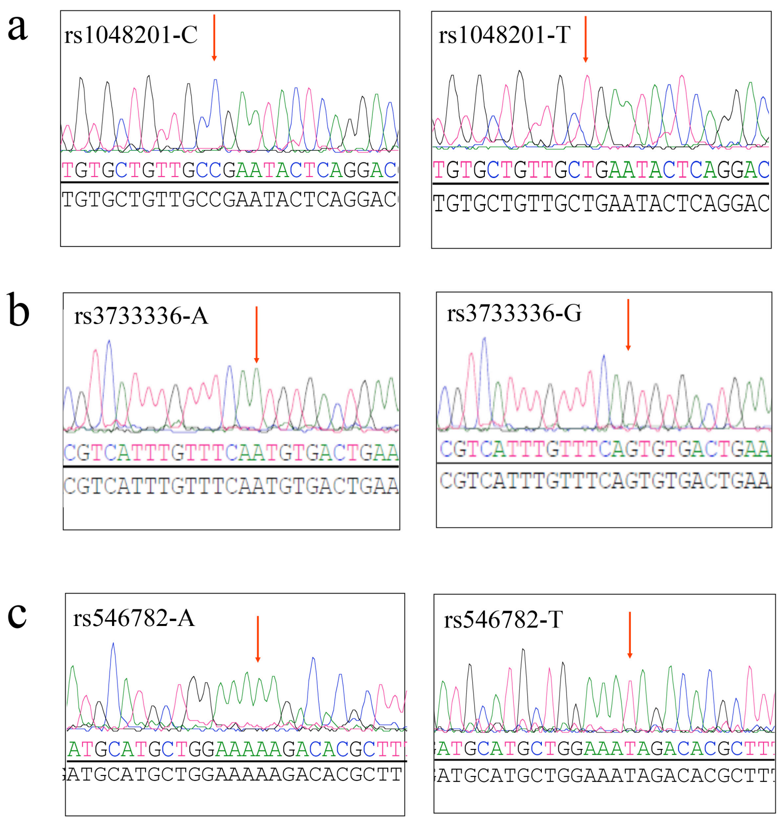

Figure S4. Diagram of three fragments and the mutations

(a) Result of sequencing showed that 3'- UTR fragment containing rs1048201 C allele or T allele was successfully synthesized and inserted into psiCHECK^{TM}-2 vector.

(b) 3'-UTR fragment of *FGF5* containing A allele or G allele of rs3733336.

(c) 3'-UTR fragment of *FGF9* containing A allele or T allele of rs546782.
